# Flavonoid Stability and Biotransformation in Agricultural Soils: Effects of Hydroxylation, Methoxylation, and Glycosylation

**DOI:** 10.1021/acs.jafc.5c02814

**Published:** 2025-06-02

**Authors:** Richard Gruseck, Thilo Hofmann, Michael Zumstein

**Affiliations:** † Division of Environmental Geosciences, Centre for Microbiology and Environmental Systems Science, 27258 University of Vienna, Vienna 1090, Austria; ‡ Doctoral School in Microbiology and Environmental Science, University of Vienna, Vienna 1090, Austria

**Keywords:** biopesticide, sustainable agriculture, structure−stability relationship, soil half-life

## Abstract

Stricter pesticide regulations are increasing the demand for environmentally acceptable alternatives with flavonoids seen as promising candidates for use as biopesticides. However, the current limited understanding of the environmental fate of flavonoids in soils restricts their assessment as active pesticide ingredients. To address this knowledge gap, we conducted laboratory incubation experiments with LC-MS-based quantification to determine the half-lives of 18 structurally related flavonoids in three agricultural soils. Hydroxylated flavonoids were rapidly transformed (*t*_1/2_: 3–12 h), while methoxylated derivatives exhibited substantially longer half-lives, which increased with the number of methoxy groups (*t*_1/2_: 5–460 h). Glycosylated flavonoids were primarily transformed into their aglycones (*t*_1/2_: 0.5–5 h). Incubation experiments with autoclaved soil indicated that biotic processes primarily catalyzed the observed transformations. All trends were consistent across different soil types and pH values. This study provides a comprehensive overview of flavonoid stability in agricultural soils, enhancing our understanding of their potential as alternative pesticides.

## Graphical abstract

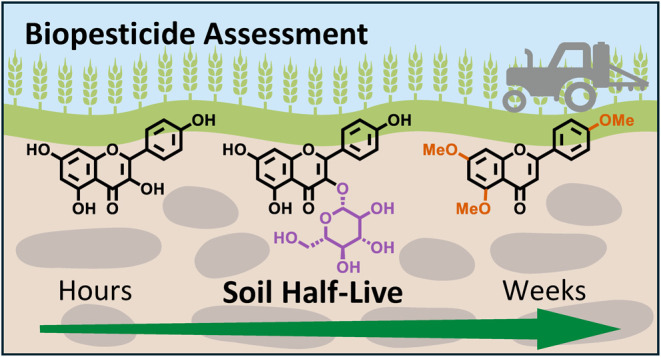

## Introduction

Increasingly stricter regulations have led to the ban or phase-out of many pesticides, with additional pesticides expected to be withdrawn in the near future.^1,2^ To mitigate the effects of pesticide restrictions, alternatives such as integrated pest management practices or more environmentally acceptable pesticides are urgently needed.^3,4^ Biopesticides, which include naturally occurring chemicals, microorganisms, and (according to some definitions) plant-incorporated protectants, are promising in this context. Several countries are currently simplifying the registration process for biopesticides due to their presumed low toxicity to nontarget organisms and rapid biodegradability.^1,4–6^ While such presumed advantages are frequently mentioned, they have yet to be conclusively demonstrated for several classes of biopesticides.

Flavonoids are secondary plant metabolites characterized by a common C6–C3–C6 backbone. In addition to their well-established roles in plants, e.g., in stress response and as signaling compounds, they are increasingly recognized as a promising class of biopesticides.^7–11^ Flavonoids can be classified into subgroups based on their chemical structure (e.g., position of the B-ring, presence of a C2–C3 double bond). Additional structural variations, including hydroxylation, methoxylation, and glycosylation, contribute to the immense diversity of flavonoids, with over 6000 known structures.^12,13^ Importantly, flavonoids can be sustainably sourced from agricultural waste products such as citrus peels and fruit kernels.^5^

The potential of flavonoids as biopesticides has recently been highlighted.^5,14^ For example, a literature review identified more than 200 studies in which flavonoids were sourced directly from plant materials as mixture or purified compounds, characterized, and evaluated for their pesticidal properties.^5^ This review further emphasized that flavonoids from all subgroups and with all aforementioned structural variations are being considered for use as biopesticides, especially as insecticides and fungicides.^5^ Another review focused on the insecticidal properties of flavonoids against several food pests, highlighting their diverse modes of action and concluding that flavonoids have significant potential as bioinsecticides.^14^ However, along with other reviews, they also highlighted the existing knowledge gaps regarding environmental fate of flavonoids in soil.^5,14–16^ The possible sourcing of flavonoids from waste products implies application to soils as mixtures, which further highlights the need to understand the effect of the flavonoid structure on their environmental fate.

The knowledge gap is supported by the small number of published articles on the fate of flavonoids in soil.^17–21^ One study reported that a range of flavonoids (e.g., quercetin-glc-rha, genistein, and naringenin) had half-lives of a few days. Another study reported half-lives for apigenin and kaempferol derivatives of several months.^17,18^ Although these studies provided an important foundation for future studies, the small number of flavonoids investigated did not allow conclusions on how the fate of flavonoids in soils is influenced by their chemical structure. A systematic study of how structural variations (i.e., hydroxylation, methoxylation, and glycosylation) affect the soil half-lives of flavonoids is still missing. These variations may also influence the transformation pathways of flavonoids in soil. Previously reported transformation pathways of flavonoids by plants and soil microbes include reactions such as hydroxylation, de/methoxylation, de/glycosylation, ring cleavage, and sulfation.^22–24^ However, these reactions are primarily studied in isolated strains for the industrial production of bioactive flavonoids, and little is known about the specific transformation reactions that predominate in soil.^23^

The objective of this study was to investigate how structural variations of flavonoids (specifically, hydroxylation, methoxylation, and glycosylation) affect their stability in agricultural soils. To achieve this, we carefully selected 18 flavonoids (Figure 1) and performed incubation experiments with three standardized soils that had different soil textures and soil pHs. Flavonoids extracted at predefined time points during soil incubations were quantified with liquid chromatography coupled to high-resolution mass spectrometry (LC-HRMS), and soil half-lives were determined. By conducting incubation experiments with autoclaved soils, the contributions of biotic and abiotic processes to the detected reactions were assessed. This study advances our understanding of flavonoid persistence in soils and thus provides valuable insights for the development of sustainable pesticide alternatives.

**Figure 1:**
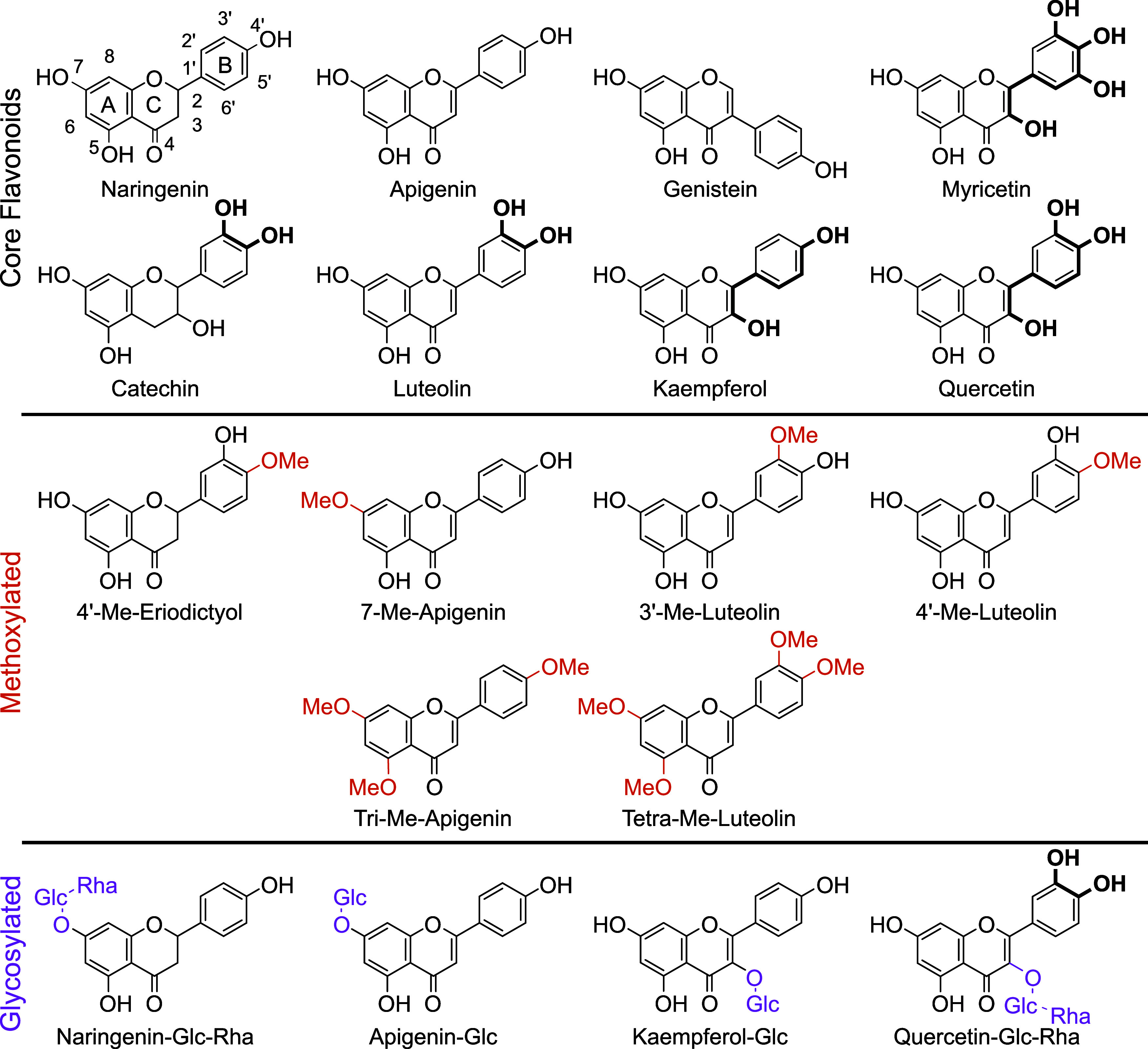
Structures and names of the investigated flavonoids. IUPAC nomenclature is added to naringenin. Conjugated hydroxyl groups that can be oxidized to quinoids are shown in bold. Methoxy groups are marked in orange, and glycosyl groups are marked in purple. Note: flavonoids grouped according to Schnarr et al., who categorized flavonoids by their core structure.^5^ Methoxylated and glycosylated flavonoids are derivatives of these core structures and are named accordingly.

## Materials and Methods

### Study Design

To establish structure–stability relationships of flavonoids in soil, we selected 8 core flavonoids as well as 6 methoxylated and 4 glycosylated derivatives of these core flavonoids (Figure 1). These flavonoids encompass most subgroups, including flavanones, flavanols, flavones, flavonols, and isoflavonoids (Figure SI 1). The core flavonoids contain between 3 and 6 hydroxy groups, and half of them include a catechol group, which can significantly influence the sorption behavior and transformation kinetics in soil.^25^ The selected core flavonoids represent 25% of all flavonoids identified in a review of over 200 publications on flavonoids as biopesticides and half of flavonoids reviewed for their insecticidal activity.^5,14^ The selected methoxylated flavonoids are mainly derivatives of apigenin and luteolin, either single or fully methoxylated. The glycosylated derivatives include either glucose, the most common glycosidic group of flavonoids, or a disaccharide consisting of rhamnose and glucose in the most common C3 and C7 positions.^26^ We focused on methoxylated and O-glycosylated derivatives, as they are the most prevalent in nature, and therefore have not included C-methylated or C-glycosylated derivatives.^13,23^

To test the effect of soil parameters on flavonoid soil half-life, we selected three soils from the “Landwirtschaftliche Untersuchungs und Forschungsanstalt (LUFA) Speyer”: Soil 2.2, 5M, and 6S (Table SI 1). These soils were selected for their varying soil textures (from sandy loam to clay) and pH (from 5.5 to 7.5), as both factors strongly influence microbial composition and abundance.^27,28^

### Chemicals and Materials

All solutions were prepared with ultrapure water (ELGA PURELAB Chorus, 0.055 μS). Catechin ((+)-catechin hydrate, 95%), myricetin (97%), quercetin (95%), kaempferol (95%), luteolin (97%), genistein (96%), apigenin (95%), 3′-Me-luteolin (chrysoeriol, 99%), 4′-Me-luteolin (diosmetin, 98%), 7-Me-apigenin (4′-5-dihydroxy-7-methoxyflavone also named genkwanin, 97%), Tri-Me-apigenin (5,7,4′-trimethoxyflavone), Tetra-Me-luteolin (3′,4′,5,7-tetramethoxyflavone, 97%), kaempferol-glc (kaempferol-3-O-glucoside, 99%), apigenin-glc (apigenin-7-O-glucoside), and quercetin-glc-rha (rutin, 95%) were purchased from abcr. Naringenin ((±)-naringenin, ≥95%) and naringenin-glc-rha (naringin, ≥95%) were purchased from Sigma-Aldrich. Acetonitrile (HPLC Gradient grade, ≥99.9%), methanol (HPLC grade, ≥99.8%), acetone (Extra Pure), and formic acid (LC/MS grade, ≥99.0%) were purchased from Fisher. Methanol (hypergrade for LC-MS, Merck), 15 and 50 mL centrifuge tubes were purchased from VWR. 2 mL centrifuge tubes (Safe-Lock tubes) were purchased from Eppendorf.

Stock solutions of flavonoids (each 1 mM) were prepared in 70% acetone (aq). Flavonoids were split into three stock solutions to mitigate the poor solubility of flavonoids. Stock solution A consisted of short-lived core flavonoids, namely, catechin, luteolin, kaempferol, quercetin, and myricetin; stock solution B consisted of all methoxylated flavonoid and the more stable core flavonoids apigenin, genistein, and naringenin; and stock solution C consisted of all glycosylated flavonoid and 4′-Me-luteolin. All stock solutions were stored at −18 °C. Calibration series were prepared from these stock solutions in 30% methanol (aq) with 0.1% formic acid covering a concentration range of 5–2000 nM.

### Soil Incubation Experiments

Agricultural soils were obtained from LUFA (Table SI 1) and maintained either as a meadow or left uncultivated without any fertilization or pesticide inputs for the past five years. The soils were stored, air-dried, and sieved to 2 mm by LUFA. After arrival, they were stored at 4 °C for up to 4 months. The soil was prewetted to 50% of their maximum water holding capacity at least 3 days prior to the start of incubations. For autoclaved controls, a portion of the prewetted soil was autoclaved at 121 °C for 30 min using a Sanoclav LaM-3–20-MCS-J autoclave.

Soil incubations were conducted in triplicates for each flavonoid stock solution. 10 μL of a flavonoid stock solution was added to one-quarter of the soil (1.25 g dry weight basis) in a 50 mL centrifugation tube. The soil was briefly mixed, and the acetone from the stock solution was allowed to evaporate for at least 20 min. Subsequently, the rest of the soil (3.75 g on a dry weight basis) was added and mixed. For recovery time points, the flavonoid solutions were directly spiked to 5 g of soil (dry weight basis). The soil samples were stored at room temperature, aerated twice a week for 1 h, and rehydrated to compensate for water loss due to evaporation. Acetone was selected as the solvent for the flavonoid stock solutions, and flavonoids were spiked initially to a portion of the soil to minimize effects on the soil microbiome, as described in previous studies.^29,30^ The applied flavonoid concentration in soil was between 0.5 and 1.25 mg/kg, which is in the lower range of pesticide application concentrations.^31^ To confirm that no detectable background levels of flavonoids were present in the tested soils, we additionally analyzed unspiked soil samples.

Flavonoids were extracted from the soil at 0, 0.5, 1, 2, and 4 h and 1, 2, 3, 5, 7 (8 for Soil 2.2), 10, 14 (15 for Soil 2.2), and 20 days. Stock A was sampled only up to the end of day 1. To extract flavonoids from the soil, we adapted a method from Andersen et al.^32^ 10 mL of acetonitrile was added to the soil, and the sample was vortexed and mixed on a horizontal shaker (Universal Shaker SM-30-B, Edmund Bühler GmbH) at 150 rpm. After 30 min, the suspension was centrifuged at 7000*g* for 5 min. Five mL of the supernatant were transferred into a 10 mL centrifuge tube, while the remaining supernatant was discarded. The extraction was repeated with 70% methanol (aq), and the combined samples were stored at −18 °C. For analysis, a 1.6 mL aliquot was transferred to a 2 mL tube and dried in a vacuum concentrator (Eppendorf Concentrator plus) at 45 °C for 3 h. The dried sample was resolubilized in 800 μL of 30% methanol (aq) containing 0.1% formic acid and vortexed. After centrifugation at 20,000*g* for 2 min, the sample was transferred to HPLC vials and measured immediately.

### HPLC-HRMS Analysis

We analyzed flavonoids using high-performance liquid chromatography (Vanquish Horizon UHPLC System, Thermo Fisher) coupled with a high-resolution mass spectrometer (HRMS) (Orbitrap Exploris 240, Thermo Fisher). Separation was achieved using a phenyl-hexyl column (Acquity Premier CSH, 1.7 μm, 2.1 × 100 mm, Waters), with an injection volume of 10 μL, a flow rate of 0.4 mL/min, a column compartment temperature of 40 °C, and the following eluents: (A) Purified water with 0.1% (v/v) formic acid and (B) methanol with 0.1% (v/v) formic acid. The eluent gradient was as follows: 0–3 min: 20% B, 3–16 min: 20–95% B, 16–18.5 min: 95% B, 18.5–19 min: 95–20% B, 19–22 min: 20% B. The MS parameters were set as follows: ion source: heated electrospray ionization, sheath gas: 50, aux gas: 10, sweep gas: 1, ion transfer tube temperature: 320 °C, vaporizer temperature: 350 °C, spray voltage: +3500 V and −2500 V, EASY-IC: start of the run, MS full-scan: range: 100–650 *m*/*z*, resolution: 90,000 (single polarity) or 60,000 (polarity switching), AGC target: Standard, Maximum IT: AUTO, MS/MS acquisitions: Top 3, resolution 22,500, AGC target: 50,000, Maximum IT: AUTO, isolation window: 1.0 *m*/*z*, NCE: 35, 55, 70 (negative mode) and 30, 50, 70 (positive mode). Soil incubation samples with stock solutions A and B were measured in negative mode, while stock solution C was measured in positive and negative (switch) mode.

### Data Analysis

HRMS data were analyzed using Skyline software (Version 22.2.0.527). For quantification, calibration curves, either linear or quadratic with a weighting factor of (1/x), were created. The calibration range was selected so that all calibration solutions had an accuracy between 80 and 120%. The limit of quantification (LOQ) was defined as the lowest-used calibration sample, and LOQ values were determined separately for each measurement sequence (Table SI 2). Instrument drift was corrected by using repeated injections of a single calibration solution throughout the measurement sequence. Flavonoids were identified based on exact mass (±3 ppm), retention time, and MS/MS spectra (Table SI 3). Soil half-lives were determined by assuming first-order kinetics. A linear fit was applied to the logarithmic flavonoid concentration over time (t), where the negative slope represents the reaction rate coefficient (k):

$$\ln\left([f\mathrm{lavonoid}{]}_{t}\right)=-k\times t+C$$

The linear fit was applied until the flavonoid concentration decreased to 5% of the initial concentration. If the concentration remained above 95% of the initial concentration during the first 4 h of incubation, a lag phase was reported, and the linear fit was delayed accordingly. Half-lives were subsequently calculated by using the following equation:

$$t_{1/2}=\ln\left(2\right)/k$$

## Results & Discussion

### Core Flavonoids Are Rapidly Transformed Or Not Recoverable

Incubation experiments with core flavonoids showed distinct differences between flavonoids that can be oxidized to quinoids and those that cannot. The core flavonoids that cannot be oxidized to quinoids (i.e., naringenin, apigenin, and genistein) were extracted from soils with recoveries between 64 and 108% (Figure SI 2). These flavonoids were transformed in a few hours (*t*_1/2_ < 12 h), with similar values across all three soils (Figures 2 and SI 3). We note that the observed half-lives may have been influenced by our experimental setup, which involved incubating up to eight flavonoids in combination. However, this approach reflects practical applications in which flavonoids are typically introduced as complex mixtures. In autoclaved soils, the transformation was significantly slower, highlighting the contribution of the biotic transformation. Naringenin had consistently shorter half-lives than apigenin and genistein, while apigenin and genistein had similar half-lives (Table SI 4). This suggests that the absence of a C2–C3 double bond reduces the half-life of flavonoids, while the position of the B-ring has a minimal influence on the soil half-life. Both structural variations are often mentioned as important predictors for bioactivity of flavonoids.^13^

**Figure 2:**
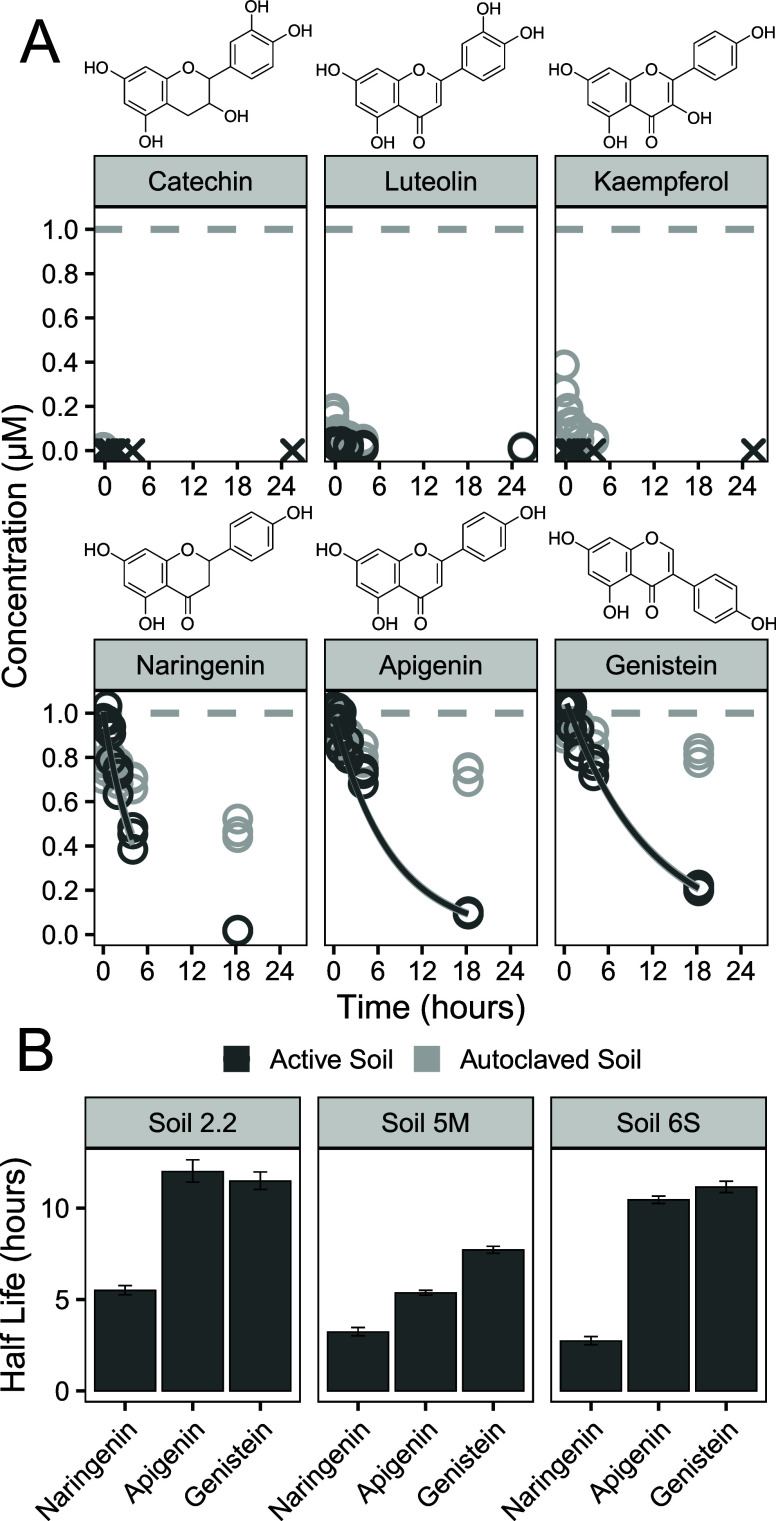
(A) Concentration of core flavonoids during their incubation in Lufa 5M soil. Concentrations below the LOQ are marked with an “X”. The gray horizontal dashed lines indicate the spiked concentration. The dark gray solid lines indicate the fitted first-order kinetics (including data points >5% of the initial concentration). (B) Soil half-lives of core flavonoids derived from fitted first-order kinetic. Error bars represent the standard error of the fit.

For the core flavonoids that could potentially be oxidized to quinoids (i.e., quercetin, myricetin, catechin, luteolin, and kaempferol), no soil half-lives could be determined. Quercetin and myricetin already degraded rapidly in calibration solutions (30% MeOH (aq)), rendering accurate quantification impossible (Figure SI 4). Given this rapid degradation in aqueous solutions, we also anticipated rapid degradation in soil and excluded quercetin and myricetin from further soil experiments. Catechin, luteolin, and kaempferol were stable in calibration solutions but showed poor recovery (<5%) from active soil (Figure 2). In autoclaved soils, recovery was higher than in active soils, with a trend that recovery increased with a higher sand content of the soil (Figure SI 2). This low recovery is consistent with previous studies that also failed to recover spiked flavonoids susceptible to oxidation into quinoids.^17,32^

To investigate the transformation pathway of these core flavonoids, we searched for oxidative transformation products, as oxidation is commonly observed for many flavonoids across various media.^33–36^ For myricetin and quercetin, we identified one transformation product each in the calibration solution that accumulated over time. We identified these two products as benzofuranone derivatives, which is consistent with previous studies on flavonoid oxidation products (Figures SI 5 and SI 6).^37^ For kaempferol, we detected the respective benzofuranone only in Soil 2.2 with decreasing abundance over time (Figure SI 7). For the other flavonoids, we could not detect other common oxidative transformation products in the soil, which is probably due to further rapid transformation (Table SI 5).

### Methoxylation Increases Soil Half-Lives

To determine the effect of methoxylation on the soil half-lives of flavonoids, we incubated two apigenin derivatives and three luteolin derivatives. Methoxylated flavonoids had recoveries between 60% and 110%, with similar values in both active and autoclaved soils (Figure SI 2). Methoxylated derivatives of apigenin and luteolin had significantly longer half-lives compared to the respective core flavonoids (Figure 3 and SI 8). For apigenin, the addition of a methoxy group (resulting in 7-Me-apigenin) increased the soil half-lives (i.e., from 5.36–12.0 h to 33.5–66 h). Full methoxylation of apigenin (resulting in Tetra-Me-apigenin) further increased the half-lives to several days and in soil 6S up to weeks (i.e., 108 ± 9, 110 ± 8, and 462 ± 49 h for soil 2.2, 5M, and 6S, respectively). Moreover, the onset of transformation for Tetra-Me-apigenin did not occur directly after spiking but only after 3–8 days of incubation (Figure SI 9). For luteolin, methylation of the catechol group (resulting in 3′-Me-Luteolin and 4′-Me-Luteolin) resulted in higher recovery from active soils (i.e., 72 ± 10% compared to <5%). The half-lives of the methoxylated derivatives were between 4.9–26.7 h, which is comparable to the stability of the core flavonoids apigenin and genistein. Methylation of the 4′–OH position resulted in higher soil half-lives (6.74–26.7 h) compared to the 3′–OH position (4.9–11.1 h). As observed with apigenin, full methoxylation of luteolin significantly increased the half-lives to several days for Soil 2.2 and 5M, respectively, with a lag phase of 3 days (Table SI 4). In soil 6S, the lag phase extended to 15 days, preventing determination of a half-life (Figure SI 8). Incubation in autoclaved soils showed reduced transformation kinetics for all methoxylated flavonoids, highlighting the contribution of biotic transformation. The slow decrease in concentration over time in autoclaved samples is likely attributable to a combination of abiotic transformation and residual biotic metabolism by microorganisms that may regrow following the single autoclaving step. Future studies could help distinguish between these processes through more thorough microbial inactivation.

**Figure 3:**
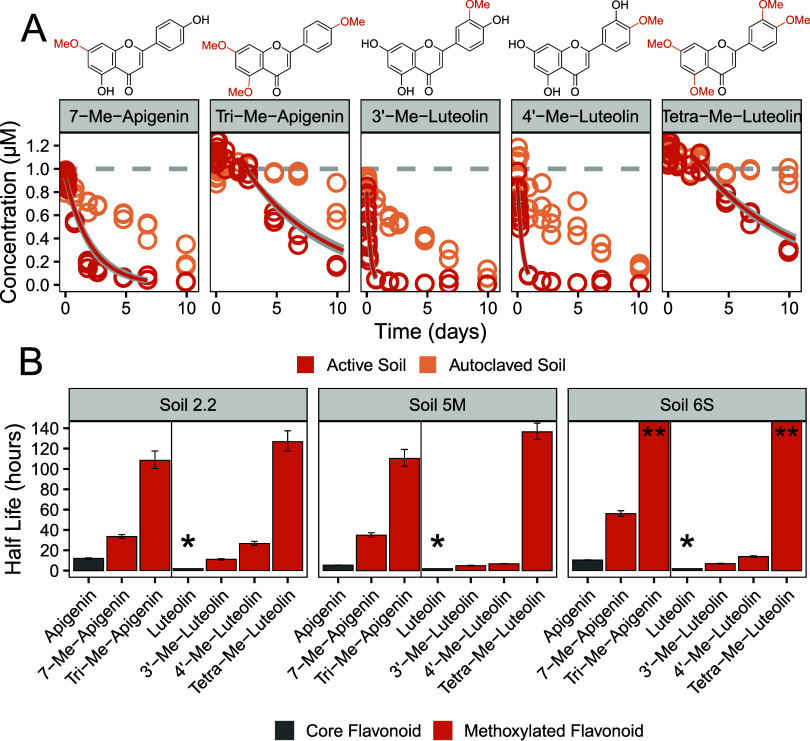
(A) Concentration of methoxylated flavonoids during their incubation in Lufa 5M soil. The gray horizontal dashed lines indicate the spiked concentration. The orange solid lines indicate the fitted first-order kinetics (including data points >5% of the initial concentration). For Tri-Me-Apigenin and Tetra-Me-Luteolin, the fitted first-order kinetics were adjusted to account for the observed lag phase, which lasted 3 days. (B) Soil half-lives of methoxylated flavonoids and their respective core flavonoid derived from fitted first-order kinetic. Error bars represent the standard error of the fit. Half-lives marked with an asterisk (*) could not be determined due to rapid removal of the flavonoid (i.e., recovery <5%). Half-lives marked with two asterisks (**) are greater than 450 h.

The addition of a 3′-methoxy group to apigenin (resulting in 3′-Me-luteolin) did not significantly change the soil half-lives (3′-Me-luteolin half-lives are 66, 91 and 93% of the half-lives for apigenin). Combined with the trend that more methoxy groups and accordingly fewer hydroxy groups increased the half-lives of flavonoids, our data suggest that the number of available hydroxy groups is a key determinant of soil half-lives. Furthermore, the removal of the C2–C3 double bond from 4′-Me-luteolin (resulting in 4′-Me-eriodictyol) drastically reduced soil half-lives, similar to the core flavonoids naringenin and apigenin (Figure SI 10).

Demethylation of polyphenols is a well-studied transformation pathway, particularly in the context of lignin demethylation.^38^ For flavonoids, demethylation was shown *in vitro*, for example, by several *aspergillus* species and lactic acid bacteria.^23,39^ However, the observed lag phase of several days for fully methoxylated flavonoids suggests the possibility that suitable enzymes are not initially present in the soil. The subsequent slow transformation kinetics indicates that potential demethylation is a slow process. Notably, the only significant differences between the three soils were observed here: in soil 6S, which has the highest clay content, the lag phase was considerably longer, and the subsequent transformation rate was slower than that in the other two soils. We detected only trace levels of demethylated products (<1% of initial Tetra-Me-luteolin and Tri-Me-apigenin area) through a high-resolution mass spectrometry suspect screening (Table SI 5). This is probably due to low steady-state concentrations of intermediates resulting from slow production rates and fast subsequent transformation rates (as observed for single methoxylated flavonoids).

### Glycosides Are Rapidly Transformed Into Their Aglycone

The effect of glycosylation on the soil half-lives of flavonoids varied depending on the structure of the aglycone (Figures 4 and SI 11). The half-lives of naringenin-glc-rha were similar to that of naringenin (Table SI 4). Naringenin was only detected partially as a transformation product (conversion rate after 4 h incubation: 19–42%). We tried to quantify the possible intermediate naringenin-glc but only detected trace levels (<1% of initial naringenin-glc-rha area, Table SI 5). In contrast, the half-lives of apigenin-glc were significantly shorter than those of apigenin. The glucose group was cleaved off with half-lives of approximately 1 h, and apigenin was found quantitatively (conversion rate after 4 h incubation: 80–93%) as a transformation product. The deglycosylation is therefore a much faster process than the subsequent transformation of apigenin. Kaempferol-glc had also half-lives of approximately 1 h. Direct comparison to the aglycone is not possible since kaempferol was not recovered (Figure SI 2), and the half-lives could not be determined. For the same reason, no aglycone of kaempferol-glc was detected. However, glycosylation of the catechol moiety in kaempferol improved the recovery and thus the stability of the flavonoid, a trend that was also observed with methoxylation of luteolin. The protection of the catechol group from luteolin resulted in half-lives up to 1 day, while the glycosylation only resulted in half-lives of around 1 h. For all glycosylated flavonoids, deglycosylation products were only identified in active soil incubations and the half-lives of glycosylated flavonoids in autoclaved soils were significantly higher (Figures 4 and SI 11). These findings indicate that the transformation of glycosylated flavonoids is driven by biotic processes. The initial abiotic decreases for kaempferol-glc and apigenin-glc are likely due to sorption.

**Figure 4:**
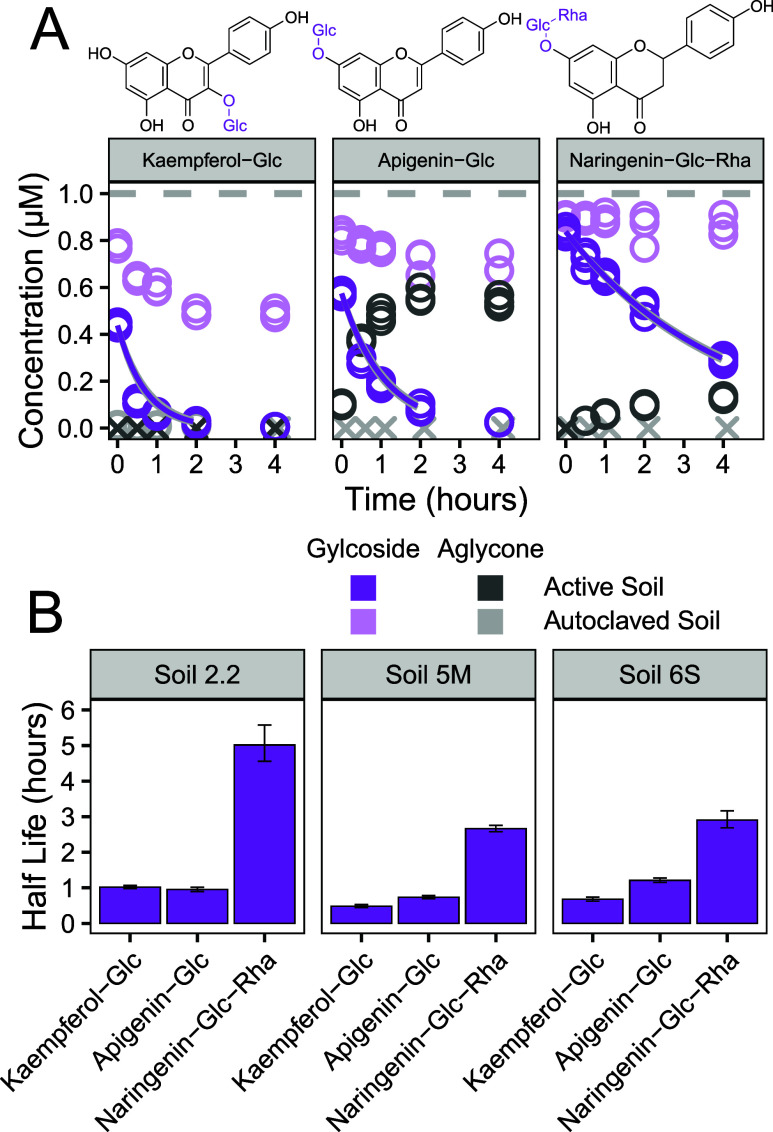
(A) Concentration of glycosylated flavonoids during their incubation in Lufa 5M soil with their transformation product. Concentrations below the LOQ are marked with an “X”. The gray horizontal dashed lines indicate the spiked concentration. The purple solid lines indicate the fitted first-order kinetics (including data points >5% of the initial concentration). (B) Soil half-lives of glycosylated flavonoids derived from fitted first-order kinetics. Error bars represent the standard error of the fit.

We suspect that the deglycosylation kinetics of flavonoids are primarily determined by the structure of the glycoside. As we have shown for apigenin-glc, glucose is rapidly cleaved, probably by abundant glucosidases in soil.^40^ Kaempferol-glc and apigenin-glc have remarkably similar half-lives (i.e., 0.48–1.02 h for kaempferol-glc and 0.74–1.21 h for apigenin-glc) and both contain a single glucose moiety. This similarity suggests that kaempferol-glc also undergoes deglycosylation similar to that of apigenin-glc. This could, however, not be proven as kaempferol itself is not stable in soil. The position of the glucose group appears to have only a minimal influence on the half-life of these glycosides, and the rapid deglycosylation of glucose moieties in all flavonoids can be expected. In contrast, naringenin-glc-rha, which contains a glucose-rhamnose disaccharide, shows significantly longer half-lives than apigenin-glc and kaempferol-glc. Moreover, the deglycosylation rate of naringenin-glc-rha is likely slower than the measured half-life implies, as less aglycone was quantified than expected in two soils (even when accounting for the simultaneous transformation of naringenin, Figure SI 11). This discrepancy indicates that naringenin-glc-rha undergoes an additional transformation pathway that does not involve deglycosylation. The expected deglycosylation pathway for naringenin-rha-glc involves naringinase, a multienzyme complex comprising α-rhamnosidase and β-glucosidase, which can sequentially cleave off both sugar moieties.^41^ This enzyme class, which is present in soil microorganisms, appears to be less active than glucosidases across all tested soils.^41^

In summary, our results highlight that small structural variations of flavonoids can dramatically alter their soil stability, with half-lives ranging between hours and weeks. Glycosidic groups in flavonoids are rapidly cleaved in all soil types, resulting in the release of the aglycone, while methoxylation considerably enhances flavonoid stability in soil. The increased stability of methoxylated derivatives could result in persistent environmental residues, particularly in soils with a high clay content. Given that methoxylated flavonoids are found in agricultural waste products, their unintentional application alongside active ingredients is a potential concern.^5^ Therefore, further research is needed to elucidate the transformation pathways of these compounds and evaluate the bioactivities of their transformation products. To determine the practical applicability of flavonoids as biopesticides, it is essential to identify the target organisms and modes of action, followed by integration of stability data in relevant environments, such as leaf surfaces for insecticides. Furthermore, the stability of flavonoids during pesticide formulation and application warrants further investigation, particularly with respect to mixture effects, to optimize their efficacy in agricultural contexts. Beyond their potential as biopesticides, the findings of this study also offer valuable insights into related fields, including chemical ecology and plant–microbe signaling.

## Supplementary Material



## Notes

Flavonoid overview (**Figure SI 1**); flavonoids recovery from soil (**Figure SI 2**); concentration of core flavonoids during their incubation in soil (**Figure SI 3**); peak area of quercetin and myricetin in aqueous solution over time (**Figure SI 4**); high-resolution product ion spectrum of the benzofuran transformation product of quercetin (**Figure SI 5**); high-resolution product ion spectrum of the benzofuran transformation product of myricetin (**Figure SI 6**); high-resolution product ion spectrum of the benzofuran transformation product of kaempferol (**Figure SI 7**); concentration of methoxylated flavonoids during their incubation in soil (**Figure SI 8**); concentration of methoxylated flavonoids during their incubation in soil during the first 3 days (**Figure SI 9**); soil half-lives of flavonoids with and without a C2–C3 double bond (**Figure SI 10**); concentration of glycosylated flavonoids during their incubation in soil (**Figure SI 11**); soil parameters and classification (**Table SI 1**); lower limits of quantification (LLOQ) for LC-HRMS measurements (**Table SI 2**); LC-HRMS parameters of tested flavonoids (**Table SI 3**); summary of first-order kinetic fits for flavonoid soil incubation (**Table SI 4**); HRMS suspect list (**Table SI 5**) (PDF)
