# Supplementary material for: Flavonoid Stability and Biotransformation in Agricultural Soils: Effects of Hydroxylation, Methoxylation, and Glycosylation
Source: J Agric Food Chem. 2025 Jun 2;73(23):14245–52. doi: 10.1021/acs.jafc.5c02814 (PMC12164328; doi:10.1021/acs.jafc.5c02814)
Supplement: Supplementary file 1 [file jf5c02814_si_001.pdf]

Supporting Information

Flavonoid Stability and Biotransformation in Agricultural Soils: Effects of Hydroxylation, Methoxylation, and Glycosylation

Richard Gruseck[1,2], Thilo Hofmann[1], Michael Zumstein[1*]

[1]Division of Environmental Geosciences, Centre for Microbiology and Environmental Systems Science, University of Vienna, Vienna 1090, Austria

[2]Doctoral School in Microbiology and Environmental Science, University of Vienna, Vienna 1090, Austria

[*]To whom correspondence should be addressed:

E-mail: michael.zumstein@univie.ac.at

This PDF file includes:

Figure SI 1. Flavonoid overview

Figure SI 2. Flavonoids recovery from soil.

Figure SI 3. Concentration of core flavonoids during their incubation in soil.

Figure SI 4. Peak area of quercetin and myricetin in aqueous solution over time.

Figure SI 5. High-resolution product ion spectrum of the benzofuran transformation product of quercetin.

Figure SI 6. High-resolution product ion spectrum of the benzofuran transformation product of myricetin.

Figure SI 7. High-resolution product ion spectrum of the benzofuran transformation product of kaempferol.

Figure SI 8. Concentration of methoxylated flavonoids during their incubation in soil.

Figure SI 9. Concentration of methoxylated flavonoids during their incubation in soil during the first 3 days.

Figure SI 10. Soil half-lives of flavonoids with and without a C2-C3 double bond.

Figure SI 11. Concentration of glycosylated flavonoids during their incubation in soil.

Table SI 1. Soil parameters and classification.

Table SI 2. Lower limits of quantification (LLOQ) for LC-HRMS measurements.

Table SI 3. LC-HRMS parameters of tested flavonoids.

Table SI 4. Summary of first-order kinetic fits for flavonoid soil incubation.

Table SI 5. HRMS suspect list.

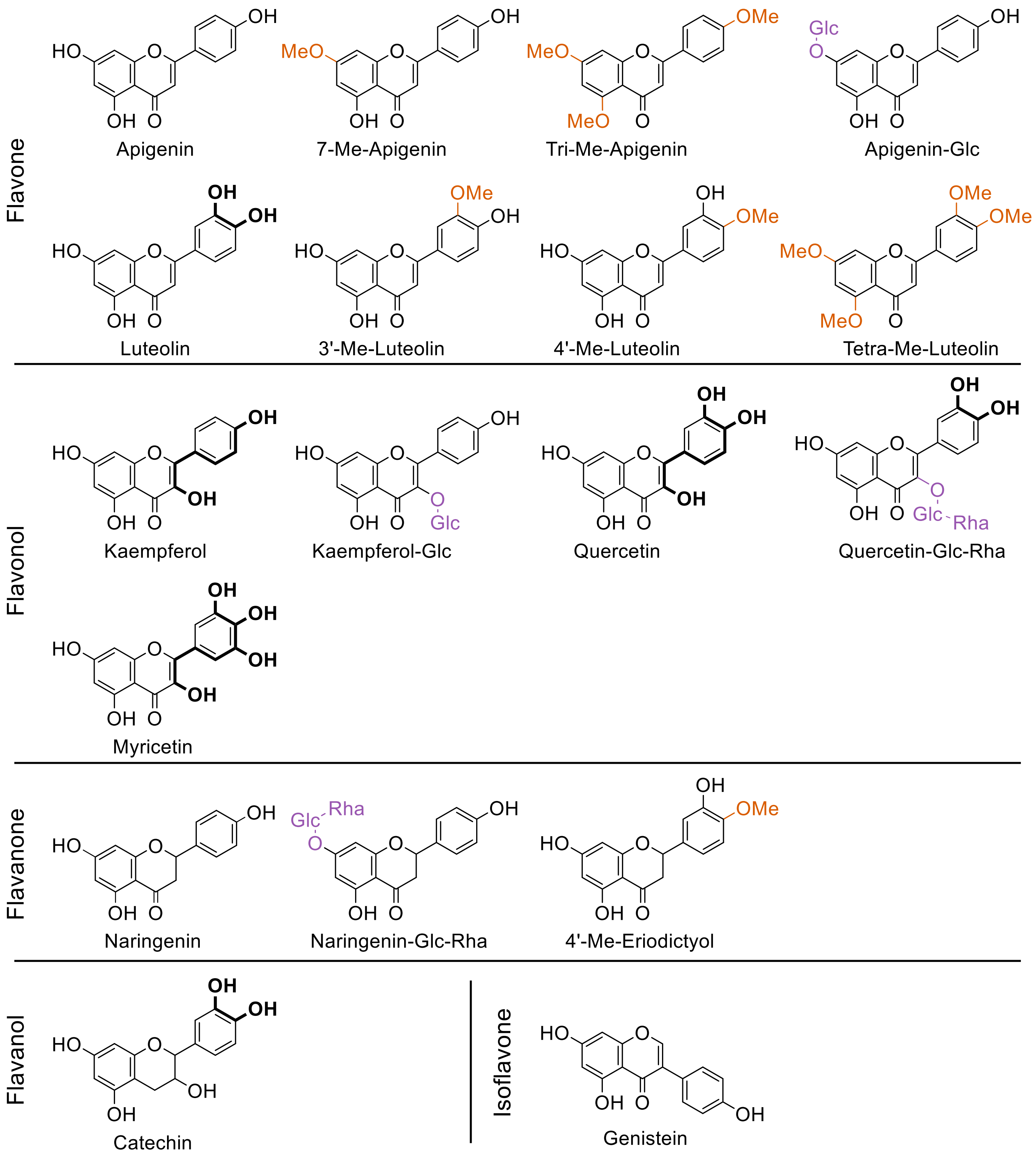

Figure SI 1: Structures and names of investigated flavonoids grouped by their flavonoid subgroup.

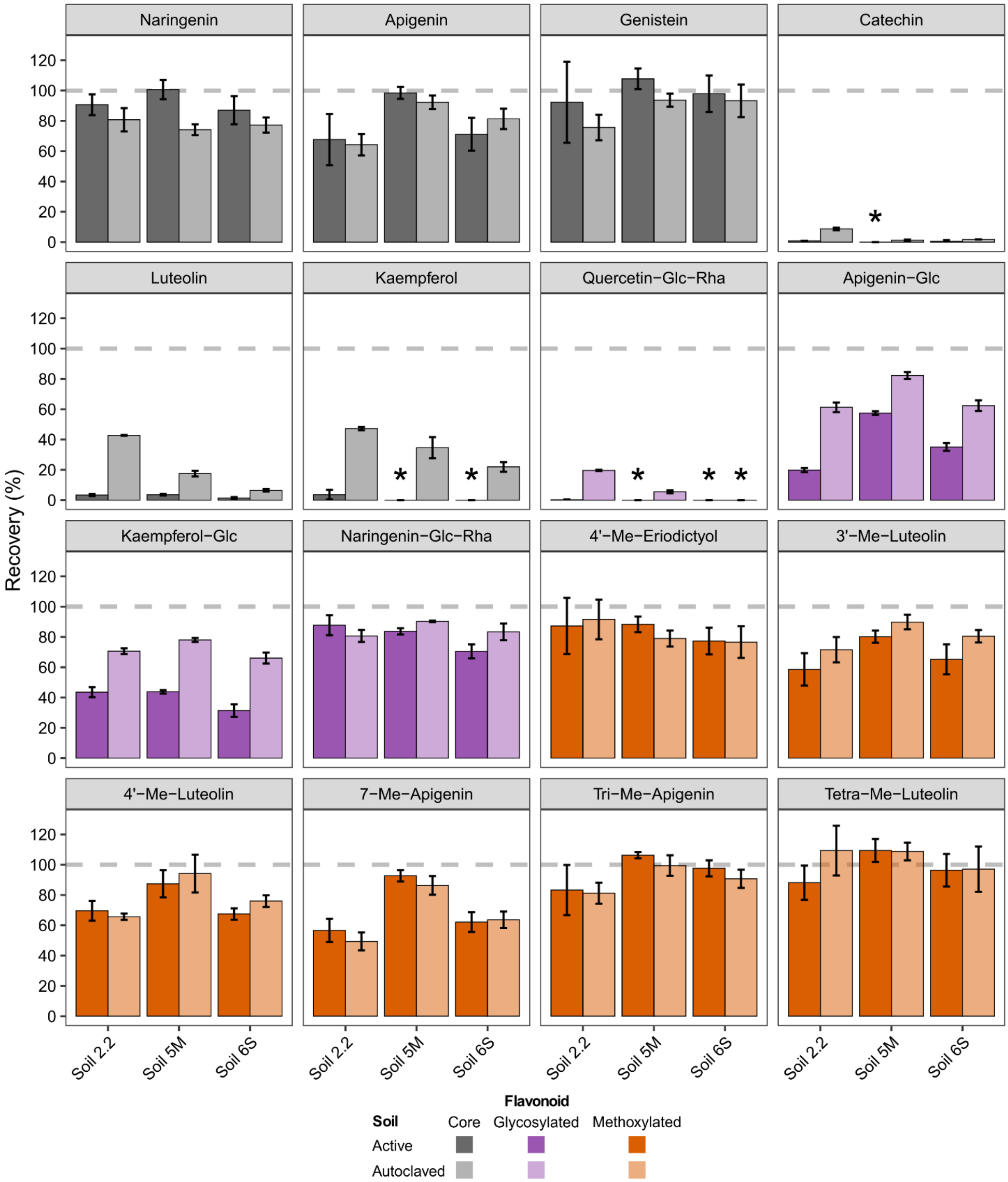

Figure SI 2: Flavonoid recovery from different soil matrices. Data points and error bars represent mean ± standard deviation of triplicate extractions. The grey horizontal dashed lines indicates 100% recovery. Recoveries marked with an asterisk (*) were below the limit of quantification.

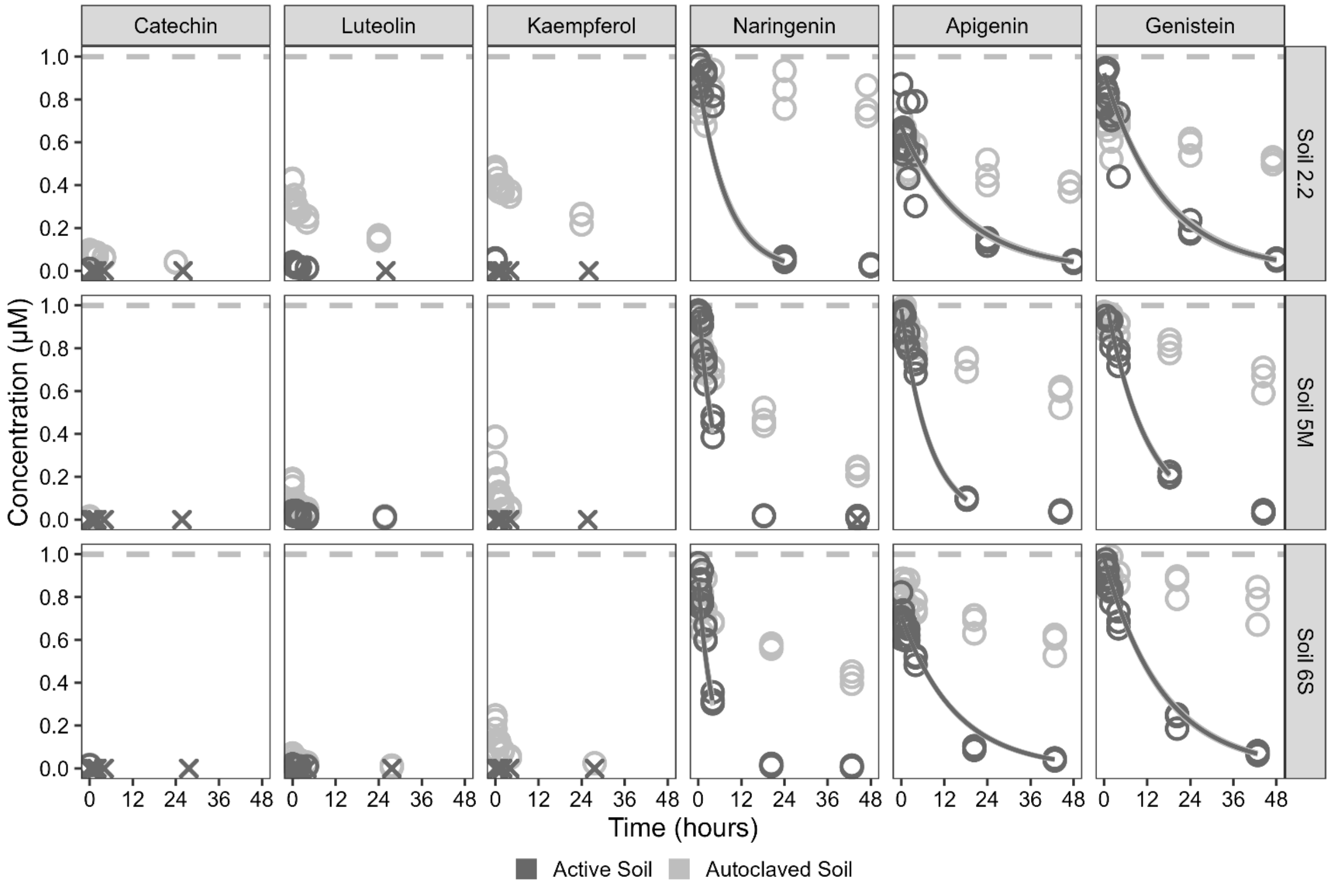

Figure SI 3: Concentration of core flavonoids during their incubation in soil. Concentrations below the LOQ are marked with a "x". The grey horizontal dashed lines indicate the spiked concentration. The dark grey solid lines indicate the fitted first-order kinetics (including data points > 5% of the initial concentration).

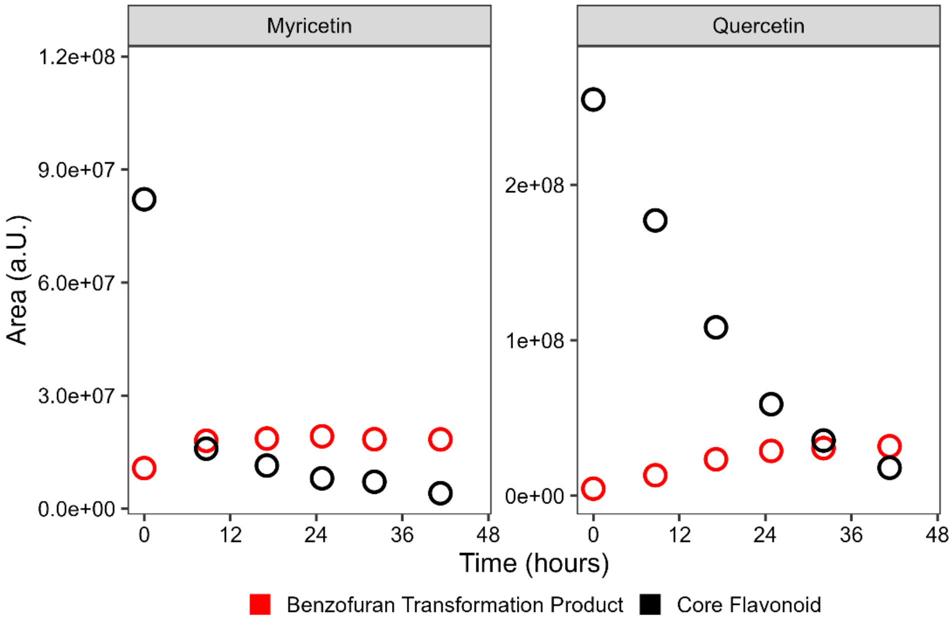

Figure SI 4: Measured peak area of a single 800 nM solution of myricetin and quercetin stored at 10° C over time with their transformation product.

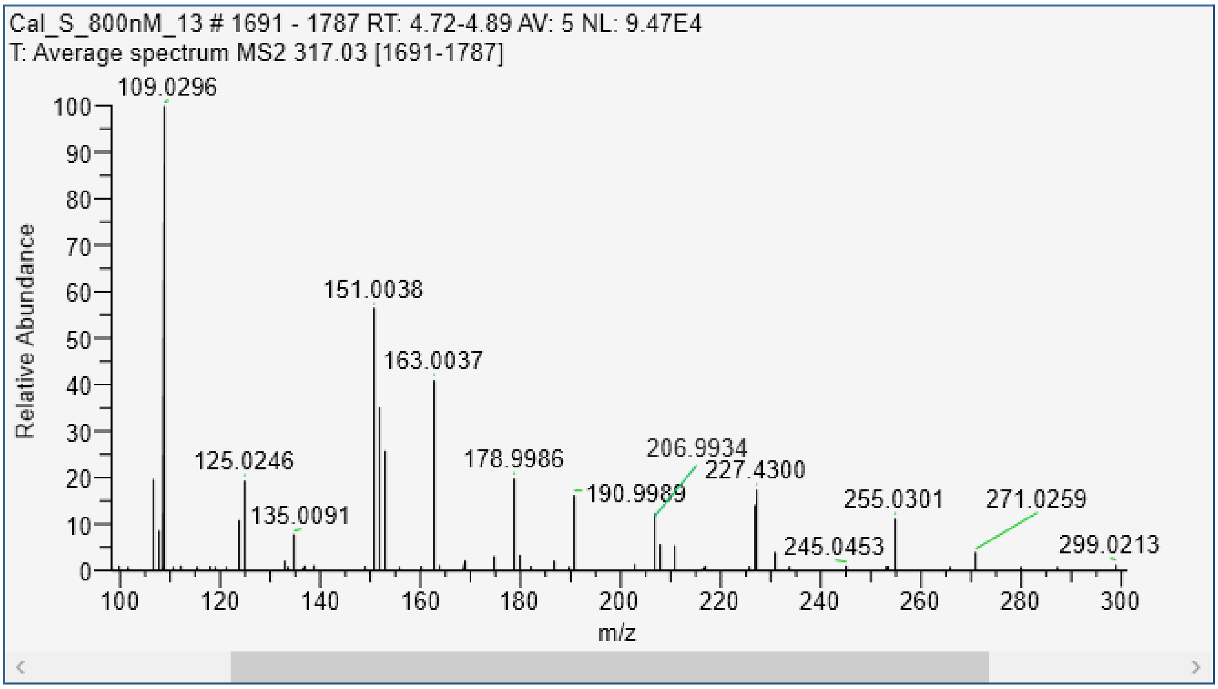

Figure SI 5: High-resolution product ion spectrum (showing a selected range of all visible peaks) of the benzofuran transformation product of quercetin (m/z: 317.0304, $C_{15}H_9O_8$, mass deviation +0.34 ppm, RT: 4.8) with the expected fragments (m/z) reported by Zhou and Sadik 2008[1]: 151, 163, 179, 191, 207, 255, 271, and 299 m/z.

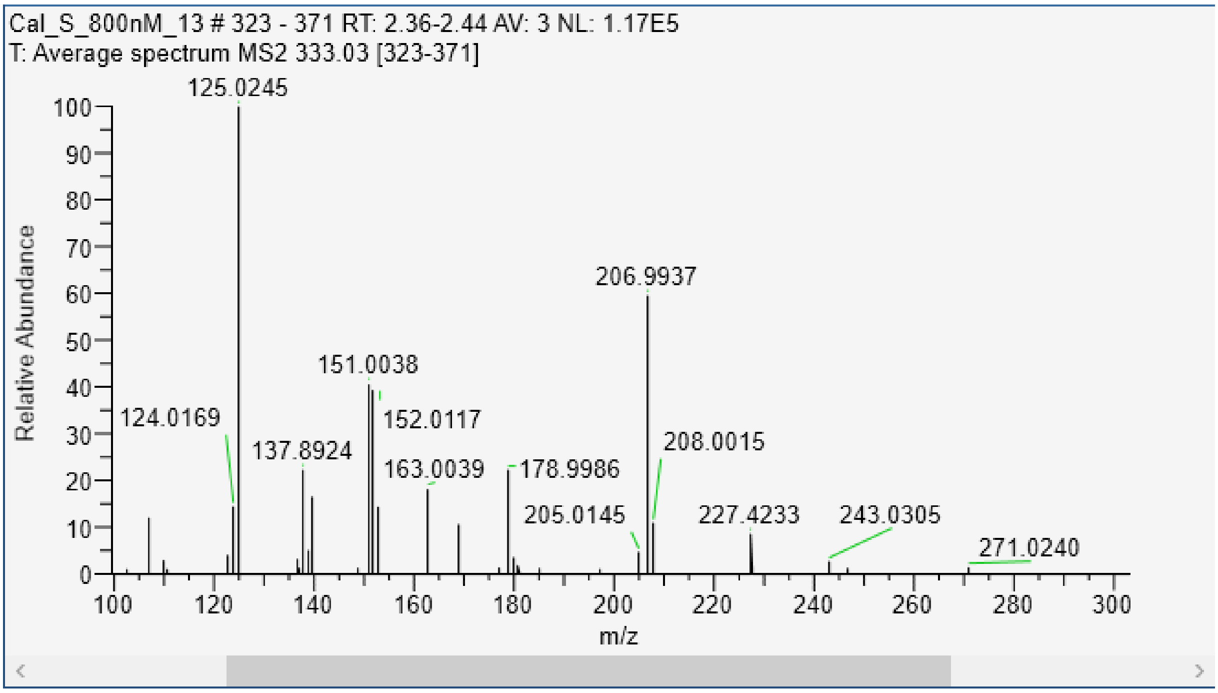

Figure SI 6: High-resolution product ion spectrum (showing a selected range of all visible peaks) of the benzofuran transformation product of myricetin (m/z: 333.0253, $C_{15}H_9O_9$, mass deviation +0.28 ppm, RT: 2.4) with the expected fragments reported by Zhou and Sadik 2008[1] for the quercetin derivative: 151, 163, 179, 207, and 271 m/z.

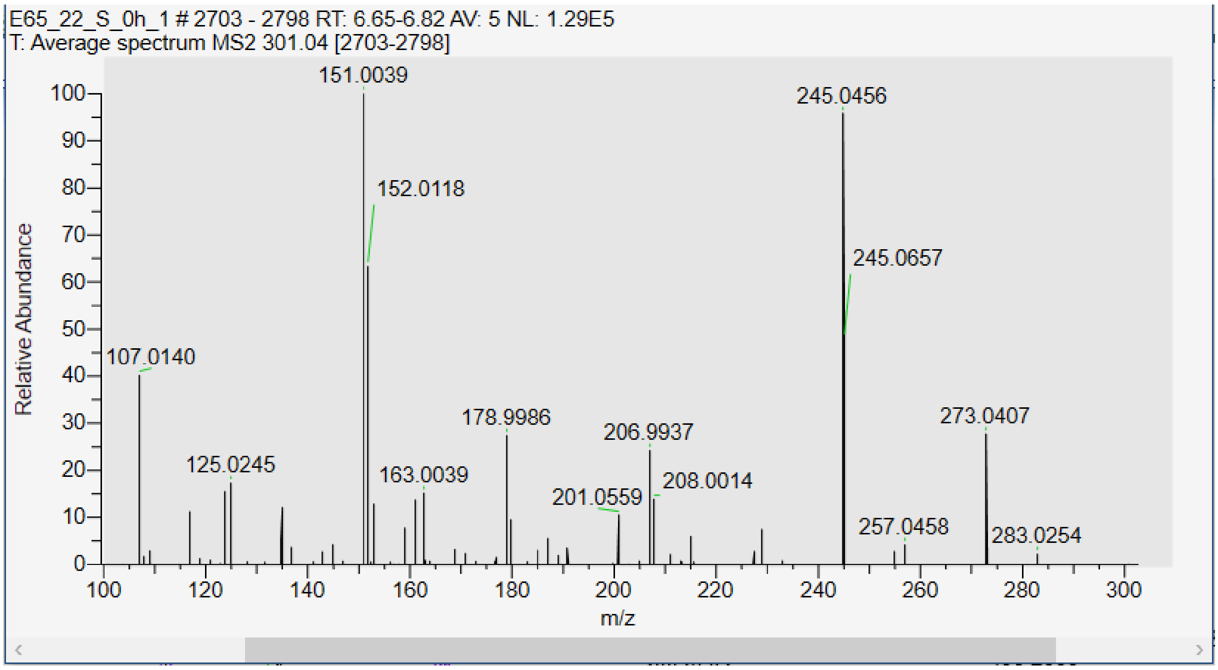

Figure SI 7: High-resolution product ion spectrum (showing a selected range of all visible peaks) of the benzofuran transformation product of kaempferol (m/z: 301.0356, $C_{15}H_9O_7$, mass deviation +0.74 ppm, RT: 6.7) with the expected fragments reported by Speisky et al., 2023[3]: 151, 207, 245, and 273 m/z.

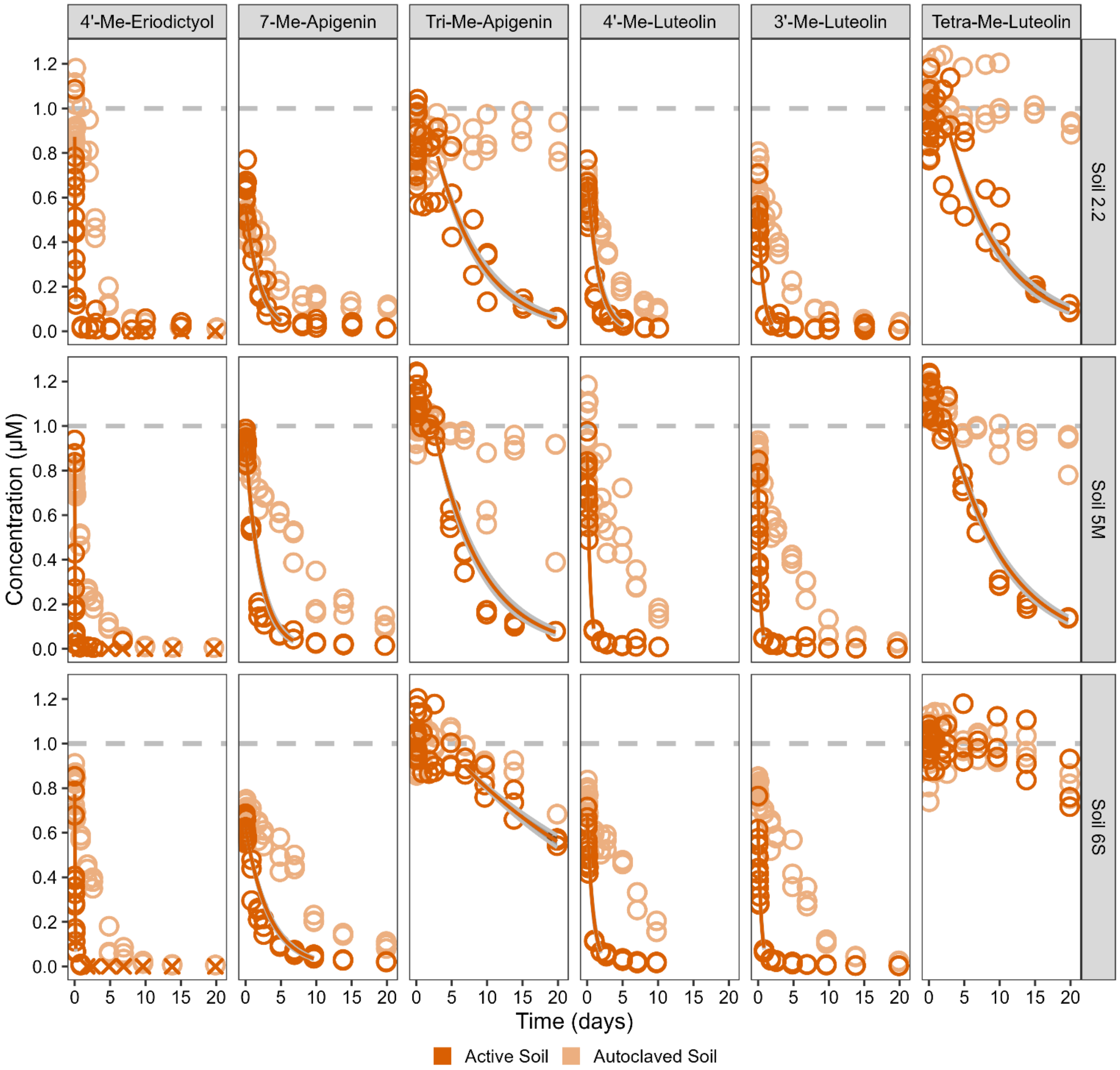

Figure SI 8: Concentration of methoxylated flavonoids during their incubation in soil. Concentrations below the LOQ are marked with a “x”. The grey horizontal dashed lines indicate the spiked concentration. The orange solid lines indicate the fitted first-order kinetics (including data points > 5% of the initial concentration). For Tri-Me-Apigenin and Tetra-Me-Luteolin, the fitted first-order kinetics were adjusted to account for the observed lag phase. For Soil 2.2 and 5M, the lag phase lasted 3 days, while for Soil 6S, it was 8 days for Tri-Me-Apigenin and 15 days for Tetra-Me-Luteolin.

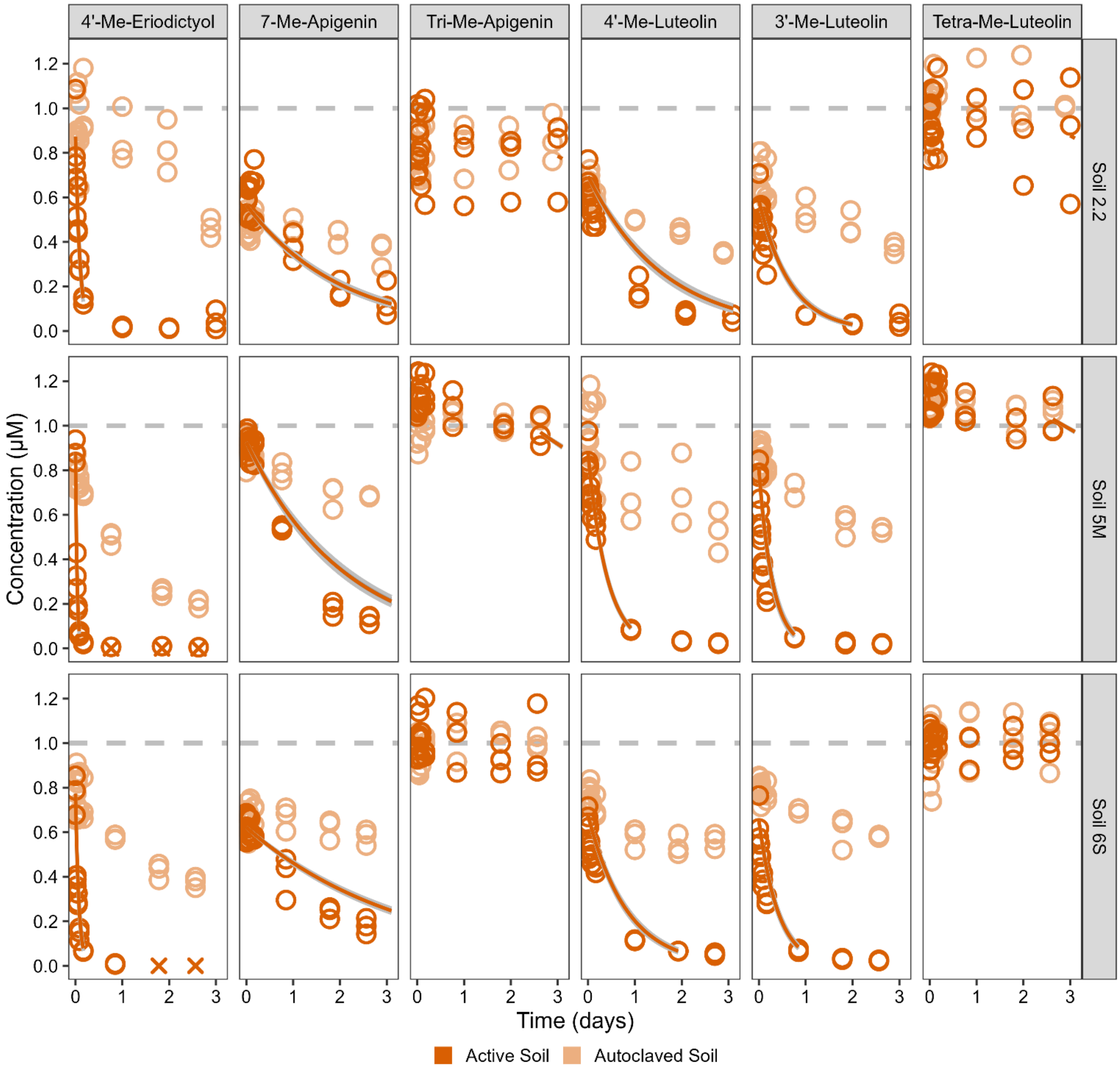

Figure SI 9: Concentration of methoxylated flavonoids during their incubation in soil over the first 3 days. Concentrations below the LOQ are marked with a "x". The grey horizontal dashed lines indicate the spiked concentration. The orange solid lines indicate the fitted first-order kinetics (including data points > 5% of the initial concentration).

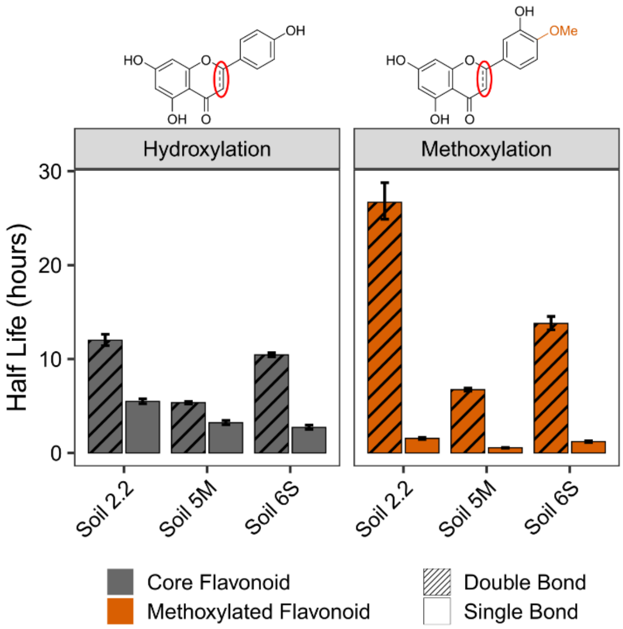

Figure SI 10: Soil half-lives of flavonoids with and without a C2-C3 double bond derived from fitted first-order kinetics from soil incubation experiments. Error bars represent the standard error of the fit.

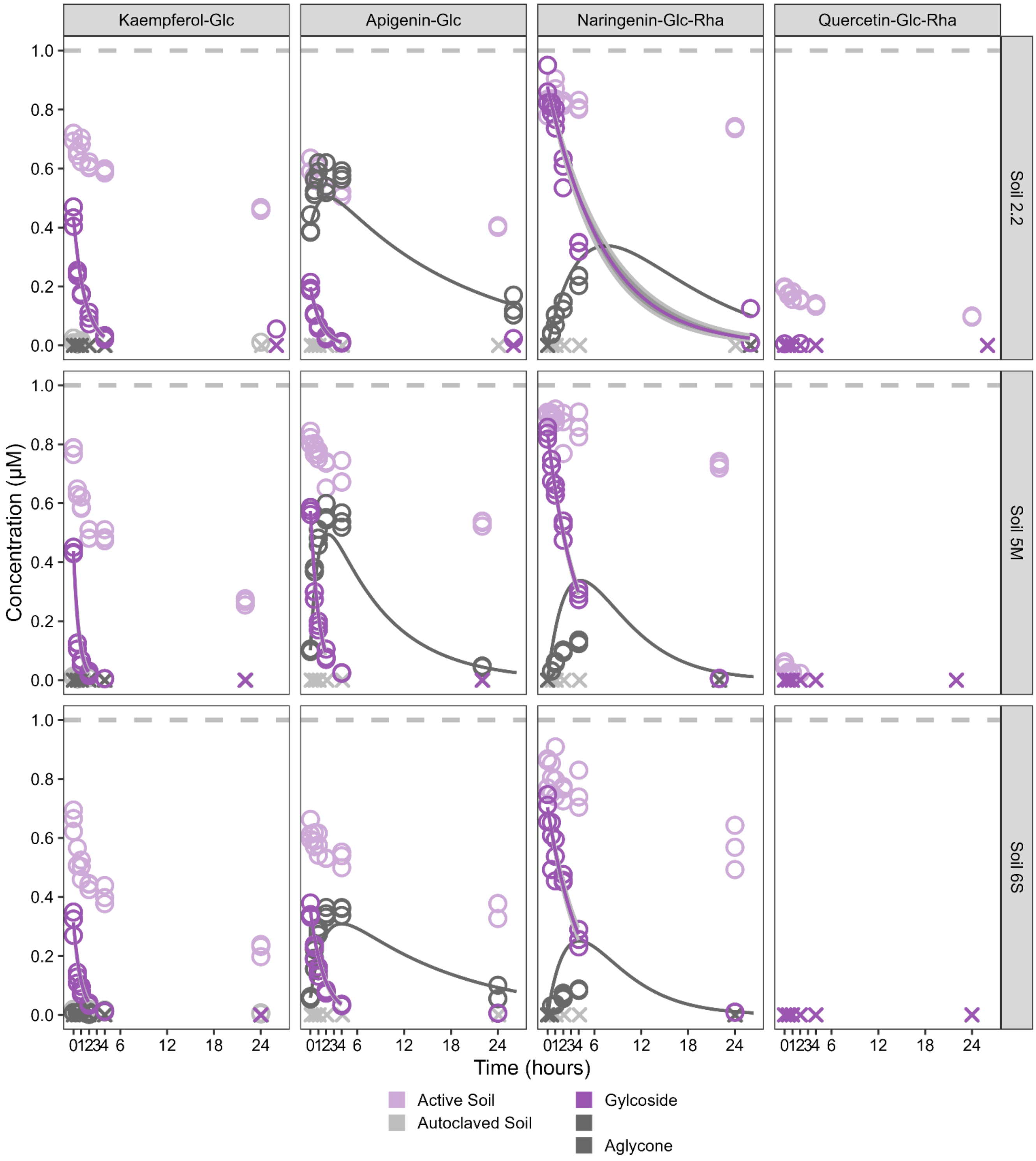

Figure SI 11: Concentration of glycosylated flavonoids during their incubation in soil with their transformation product. Concentrations below the LOQ are marked with a "x". The grey horizontal dashed lines indicate the spiked concentration. The purple solid lines indicate the fitted first-order kinetics to the glycoside data points (including data points > 5% of the initial concentration). The dark grey solid lines indicate the modelled aglycone concentration considering the production and degradation rates of the glycoside and aglycone.

Table SI 1: Soil parameters and classification adapted from “Landwirtschafltiche Untersuchungs- und Forschungsanstalt Speyer“.

Soil Parameter	Soil 2.2	Soil 5M	Soil 6S
pH value (0.01 M CaCl2)	5.60 ± 0.29	7.45 ± 0.07	7.31 ± 0.06
Organic carbon (% C)	1.82 ± 0.48	1.18 ± 0.25	1.66 ± 0.2
Cation exchange capacity (meq/100g)	9.54 ± 1.36	9.86 ± 0.78	18.67 ± 1.65
Maximum water holding capacity (g/100g)	48.9 ± 5.6	41.5 ± 3.9	47.1 ± 7.1
Particle size distribution (mm)			
<0.002	9.7 ± 1.2	13.4 ± 0.6	41.2 ± 2.3
0.002 - 0.05	16.3 ± 1.5	33.1 ± 2.4	35.9 ± 1.3
0.05 - 2.0	74.0 ± 2.2	53.4 ± 2.2	22.9 ± 1.8
Soil Type (USDA)	sandy loam	sandy loam	clay

Table SI 2: Lower limits of quantification (LLOQ) for LC-HRMS measurements, defined as the lowest calibration sample used for quantification, with values determined independently for each measurement sequence.

Flavonoid	Lower Limit of Quantification (nM)					
	Soil 2.2 Active	Soil 2.2 Autoclaved	Soil 6S Active	Soil 6S Autoclaved	Soil 5M Active	Soil 5M Autoclaved
Naringenin	5	5	5	5	5	5
Apigenin	5	5	5	5	5	5
Genistein	5	5	5	5	5	5
Catechin	5	5	5	5	5	5
Luteolin	10	5	5	5	5	5
Kaempferol	50	20	20	20	20	20
4'-Me-Eriodictyol	5	5	5	5	5	5
7-Me-Apigenin	5	5	5	5	5	5
3'-Me-Luteolin	5	5	5	5	5	5
4'-Me-Luteolin	5	5	5	5	5	5
Tri-Me-Apigenin	10	5	5	5	5	5
Tetra-Me-Luteolin	50	10	5	5	5	5
Naringenin-Glc-Rha	5	5	5	5	5	5
Apigenin-Glc	5	5	5	5	5	5
Kaempferol-Glc	5	5	5	5	5	5
Quercetin-Glc-Rha	5	10	20	20	20	20
Apigenin (Transformation Product)	20	20	20	20	20	20
Naringenin (Transformation Product)	20	20	20	20	20	20

Table SI 3: LC-HRMS parameters of tested flavonoids: chemical formula, detected precursor ion, exact mass to charge ratio (m/z), and retention times (in minutes).

Flavonoid	Chemical Formula	Precursor Ion	Exact Mass (m/z)	Retention Time (min)
Naringenin	$C_{15}H_{12}O_5$	$[M-H]^-$	271.0612	10.46
Apigenin	$C_{15}H_{10}O_5$	$[M-H]^-$	269.0455	11.16
Genistein	$C_{15}H_{10}O_5$	$[M-H]^-$	269.0455	10.51
Myricetin	$C_{15}H_{10}O_8$	$[M-H]^-$	317.0303	8.61
Catechin	$C_{15}H_{14}O_6$	$[M-H]^-$	289.0718	2.11
Luteolin	$C_{15}H_{10}O_6$	$[M-H]^-$	285.0405	10.20
Kaempferol	$C_{15}H_{10}O_6$	$[M-H]^-$	285.0405	10.73
Quercetin	$C_{15}H_{10}O_7$	$[M-H]^-$	301.0354	9.91
4'-Me-Eriodictyol	$C_{16}H_{14}O_6$	$[M-H]^-$	301.0718	10.68
7-Me-Apigenin	$C_{16}H_{12}O_5$	$[M-H]^-$	283.0612	12.68
3'-Me-Luteolin	$C_{16}H_{12}O_6$	$[M-H]^-$	299.0561	11.20
4'-Me-Luteolin	$C_{16}H_{12}O_6$	$[M-H]^-$	299.0561	11.11
Tri-Me-Apigenin	$C_{18}H_{16}O_5$	$[M+H]^+$	313.1071	12.35
Tetra-Me-Luteolin	$C_{19}H_{18}O_6$	$[M+H]^+$	343.1176	11.73
Naringenin-Glc-Rha	$C_{27}H_{32}O_{14}$	$[M-H]^-$	579.1719	8.55
Apigenin-Glc	$C_{21}H_{20}O_{10}$	$[M-H]^-$	431.0984	8.97
Kaempferol-Glc	$C_{21}H_{20}O_{11}$	$[M-H]^-$	447.0933	8.79
Quercetin-Glc-Rha	$C_{27}H_{30}O_{16}$	$[M-H]^-$	609.1461	7.91

Table SI 4: Summary of first-order kinetic fits for flavonoid soil incubation experiments, including half-lives ± standard error and the coefficient of determination (R^2). Half-lives were not calculated for flavonoids with soil recoveries below 5% and for Tetra-Me-luteolin in Soil 6S due to an extended lag phase.

**Flavonoid**	**Half-life (hours)**			**Coefficient of Determination (R^2)**		
	Soil 2.2 Active	Soil 5M Active	Soil 6S Active	Soil 2.2 Active	Soil 5M Active	Soil 6S Active
Naringenin	5.5 ± 0.3	3.22 ± 0.23	2.91 ± 0.24	0.968	0.940	0.919
Apigenin	12.0 ± 0.6	5.36 ± 0.14	10.45 ± 0.20	0.953	0.990	0.994
Genistein	11.5 ± 0.5	7.71 ± 0.19	11.1 ± 0.3	0.968	0.990	0.986
Catechin	-	-	-	-	-	-
Luteolin	-	-	-	-	-	-
Kaempferol	-	-	-	-	-	-
4'-Me-Eriodictyol	1.54 ± 0.10	0.55 ± 0.04	1.20 ± 0.09	0.947	0.959	0.933
7-Me-Apigenin	33.5 ± 1.9	34.9 ± 2.2	66 ± 3	0.925	0.896	0.931
3'-Me-Luteolin	11.1 ± 0.6	4.9 ± 0.3	6.86 ± 0.35	0.952	0.940	0.960
4'-Me-Luteolin	26.7 ± 1.9	6.74 ± 0.16	13.8 ± 0.7	0.884	0.991	0.952
Tri-Me-Apigenin	108 ± 9	110 ± 8	462 ± 49	0.916	0.917	0.900
Tetra-Me-Luteolin	127 ± 10	136 ± 8	-	0.917	0.948	-
Naringenin-Glc-Rha	5.0 ± 0.5	2.66 ± 0.09	2.91 ± 0.24	0.861	0.985	0.920
Apigenin-Glc	0.95 ± 0.06	0.74 ± 0.04	1.21 ± 0.06	0.952	0.966	0.967
Kaempferol -Glc	1.02 ± 0.05	0.48 ± 0.04	0.68 ± 0.06	0.976	0.929	0.936
Quercetin-Glc-Rha	-	-	-	-	-	-

Table SI 5. High-resolution mass spectrometry suspect list including their: chemical formula, precursor ion, exact mass to charge ratio (m/z), and references.

Flavonoid	Chemical Formula	Precursor Ion	Exact Mass (m/z)	Reference
Oxidative TP	$C_6H_6O_3$	$[M-H]^-$	125.02442	Das and Rosazza, 2006[2]
Oxidative TP	$C_8H_8O_3$	$[M-H]^-$	151.04007	Das and Rosazza, 2006[2]
Oxidative TP	$C_8H_{10}O_3$	$[M-H]^-$	153.05572	Das and Rosazza, 2006[2]
Oxidative TP	$C_{15}H_{14}O_5$	$[M-H]^-$	274.08467	Das and Rosazza, 2006[2]
Kaempferol-Benzofuranone	$C_{15}H_9O_7$	$[M-H]^-$	301.03538	Speisky et al., 2023[3]
Quercetin-Benzofuranone	$C_{15}H_9O_8$	$[M-H]^-$	317.03030	Zhou and Sadik, 2008[1]
Myricetin-Benzofuranone	$C_{15}H_9O_9$	$[M-H]^-$	333.02521	Zhou and Sadik, 2008[1]
Tri-Me-Luteolin	$C_{18}H_{16}O_6$	$[M-H]^-$	327.08741	
Di-Me-Luteolin	$C_{17}H_{14}O_6$	$[M-H]^-$	313.07176	
Di-Me-Apigenin	$C_{17}H_{14}O_5$	$[M-H]^-$	297.07685	
Tri-Me-Luteolin	$C_{18}H_{16}O_6$	$[M+H]^+$	329.10196	
Di-Me-Luteolin	$C_{17}H_{14}O_6$	$[M+H]^+$	315.08631	
Di-Me-Apigenin	$C_{17}H_{14}O_5$	$[M+H]^+$	299.09140	
Prunin	$C_{21}H_{22}O_{10}$	$[M-H]^-$	433.11402	

(1) Zhou, A.; Sadik, O. A. Comparative Analysis of Quercetin Oxidation by Electrochemical, Enzymatic, Autoxidation, and Free Radical Generation Techniques: A Mechanistic Study. *J. Agric. Food Chem.* **2008**, *56* (24), 12081–12091. https://doi.org/10.1021/jf802413v.

(2) Das, S.; Rosazza, J. P. N. Microbial and Enzymatic Transformations of Flavonoids. *J. Nat. Prod.* **2006**, *69* (3), 499–508. https://doi.org/10.1021/np0504659.

(3) Speisky, H.; Arias-Santé, M. F.; Fuentes, J. Oxidation of Quercetin and Kaempferol Markedly Amplifies Their Antioxidant, Cytoprotective, and Anti-Inflammatory Properties. *Antioxidants* **2023**, *12* (1), 155. https://doi.org/10.3390/antiox12010155.
